# Plasma exosome-derived microRNAs expression profiling and bioinformatics analysis under cross-talk between increased low-density lipoprotein cholesterol level and ATP-sensitive potassium channels variant rs1799858

**DOI:** 10.1186/s12967-020-02639-8

**Published:** 2020-12-03

**Authors:** Cheng Liu, Yanxian Lai, Songsong Ying, Junfang Zhan, Yan Shen

**Affiliations:** 1 Department of Cardiology, Guangzhou First People’s Hospital, South China University of Technology, 1 Panfu Road, Guangzhou, 510180 China; 2 Department of Gastroenterology, Guangzhou First People’s Hospital, South China University of Technology, Guangzhou, 510180 China; 3 Department of Health Management Center, Guangzhou First People’s Hospital, South China University of Technology, Guangzhou, 510180 China

**Keywords:** Exosome-derived microRNAs, Low-density lipoprotein cholesterol, ATP-sensitive potassium channels, Polymorphism, Bioinformatics analysis

## Abstract

**Background:**

Exosome-derived microRNAs (exo-miRs) as messengers play important roles, in the cross-talk between genetic [ATP-sensitive potassium channels (*KATP*) genetic variant rs1799858] and environmental [elevated serum low-density lipoprotein cholesterol (LDL-C) level] factors, but the plasma exo-miRs expression profile and its role in biological processes from genotype to phenotype remain unclear.

**Methods:**

A total of 14 subjects with increased LDL-C serum levels (≥ 1.8 mmol/L) were enrolled in the study. The *KATP* rs1799858 was genotyped by the Sequenom MassARRAY system. The plasma exo-miRs expression profile was identified by next-generation sequencing.

**Results:**

64 exo-miRs were significantly differentially expressed (DE), among which 44 exo-miRs were up-regulated and 20 exo-miRs were down-regulated in those subjects carrying T-allele (TT + CT) of rs1799858 compared to those carrying CC genotype. The top 20 up-regulated DE-exo-miRs were miR-378 family, miR-320 family, miR-208 family, miR-483-5p, miR-22-3p, miR-490-3p, miR-6515-5p, miR-31-5p, miR-210-3p, miR-17-3p, miR-6807-5p, miR-497-5p, miR-33a-5p, miR-3611 and miR-126-5p. The top 20 down-regulated DE-exo-miRs were let-7 family, miR-221/222 family, miR-619-5p, miR-6780a-5p, miR-641, miR-200a-5p, miR-581, miR-605-3p, miR-548ar-3p, miR-135a-3p, miR-451b, miR-509-3-5p, miR-4664-3p and miR-224-5p. Gene Ontology (GO) and Kyoto Encyclopedia of Genes and Genomes (KEGG) analyses were subsequently implemented to identify the top 10 DE-exo-miRs related specific target genes and signaling pathways. Only 5 DE-exo-miRs were validated by qRT-PCR as follows: miR-31-5p, miR-378d, miR-619-5p, miR-320a-3p and let-7a-5p (all *P* < 0.05).

**Conclusion:**

These results firstly indicated the plasma exo-miRs expression profile bridging the link between genotype (*KATP* rs1799858) and phenotype (higher LDL-C serum level), these 5 DE-exo-miRs may be potential target intermediates for molecular intervention points.

## Background

The low-density lipoprotein cholesterol (LDL-C), as a key atherogenic cholesterol, is an independent risk factor for atherosclerotic cardiovascular diseases (ASCVD), which become a serious public health problem [1]. The increased LDL-C plasma concentration (≥ 1.8 mmol/L) and its related ASCVD is the result of a combination of genetic and environmental factor influences. These environmental factors include lifestyle risk factors (e.g., unhealthy diet, smoking, physical inactivity and obesity, etc.), chemical and physical hazards (e.g., hyperlipidemia, hypertension and hyperglycemia, etc.), and their interactions. Comprehensive management and control of those multiple modifiable environmental risk factors is significant related to lower LDL-C level and lower cardiovascular events risk [2], but those benefits may be dampened by high genetic risk [3]. The genetic susceptibility factor, as an inherent and lifetime risk factor, is powerful independent predictor of high LDL-C level and its related ASCVD. Indeed, the ATP-sensitive potassium channels (*KATP*) variant rs1799858 was a genetic risk factor for higher LDL-C plasma concentration (≥ 1.8 mmol/L) and its related macro-/micro-vascular arteriosclerotic event risk [4]. However, the mechanism of elevated LDL-C plasma level and its induced ASCVD under specific genetic background of *KATP* variant rs1799858 remains unclear.

Co-evolution of the genetic and environment factors leads to the development of higher LDL-C serum levels and its related ASCVD. Non-coding RNA, especially the plasma exosome-derived microRNAs (exo-miRs), as the bridge between environmental factors and genetic factors, plays a critical role in this cross-talk process. Exosomes are lipid bilayer extracellular vesicles with a diameter of 30–150 nm secreted by almost all nucleated cells, which mediate cell–cell communication through their components, including microRNAs (miRs), mRNA, DNA, proteins and lipids. The miRs are small and endogenous RNAs (containing about 22–25 nucleotides), which take part in regulating multiple target genes at the post-transcriptional level. Exo-miRs are involved in kinds of physiological or pathological processes in occurrence and development of elevated LDL-C level and its related ASCVD. However, the plasma exo-miRs expression profiles under cross-talk effect between genetic and environment factors remain largely unknown. In this study, using a next-generation sequencing method, we sought to characterize the circulating exo-miRs expression profile in subjects with increased LDL-C level (≥ 1.8 mmol/L) under specific genetic background of *KATP* polymorphism rs1799858. We then performed Gene Ontology (GO) annotation and Kyoto Encyclopedia of Genes and Genomes (KEGG) pathway analysis based on predicted target genes. The top 10 DE-exo-miRs were then confirmed by individual quantitative real-time polymerase chain reaction (qRT-PCR) in 50 increased LDL-C levels (≥ 1.8 mmol/L) subjects with T-allele of rs1799858 and 50 same subjects with counterpart CC genotype. This helped us to facilitate our understanding of the molecular processes from genotype (KATP rs1799858) to phenotype (higher LDL-C serum level).

## Methods

### Study subjects

A total of 14 subjects with only increased LDL-C serum concentration (≥ 1.8 mmol/L) were recruited into the study from South China. Subjects with other types of dyslipidemia were excluded from the study, including increased levels of triglyceride (TRIG ≥ 1.7 mmol/L), total cholesterol (TC ≥ 5.2 mmol/L) or (and) apolipoprotein B (Apo B ≥ 80 mg/dL), and (or) decreased levels of high-density lipoprotein cholesterol (HDL-C < 1.0 mmol/L) and apolipoprotein AI (Apo AI < 120 mg/dL). All participants with different types of dyslipidemia were newly diagnosed according to guidelines [5]. All subjects combined with smoking, drinking, hypertension (HTN), coronary atherosclerotic heart disease (CAD), type 2 diabetes mellitus (T2D), stroke, abnormal liver function [alanine aminotransferase (ALT) or (and) aspartate aminotransferase (AST) more than 3 times upper limit of normal], abnormal kidney function [estimated glomerular filtration rate (eGFR) less than 90 ml/min·1.73 m^2^], or (and) any other medical conditions or drugs that may influence blood lipid levels were also excluded from the study. All blood biochemistry analysis was conducted on enrollment to the study by using standard analytical techniques.

### Genotyping

The extraction of genomic DNA from the whole-blood sample was performed with QIAamp DNA Blood Midi Kit (Qiagen, Dusseldorf, Germany) and stored at -20℃ according to the manufacturer’s protocol. The *KATP* single nucleotide polymorphism (SNP) rs1799858 were genotyped using MassARRAY platform (Sequenom Co., San Diego, USA) according to previously described methods [4]. The locus-specific primers were designed by Primer 5.0 (Whitehead Institute Cambridge, Massachusetts, USA) according to the gene sequence in GenBank (NC_000011.10:g.17428382C>T) as follow: (1) forward primer (5′–3′): ACGTTGGATGTGAGGCCCCGACAATCCTCC; (2) reverse primer (5′–3′): ACGTTGGATGAGTGGGTCCTCACCTCCAAA; (3) extension primer (5′–3′): GCCACTCAGGGTTGTGAACCGCAA. The accuracy of the genotypes of rs1799858 was determined 100%.

### Exosome isolation, exo-miRs sequencing and sequencing data analysis

The test process was carried out according to the following procedure: (1) Sample collection: The whole-blood samples were collected into anticoagulation vacuum tube with EDTA (3 mg/mL) on enrollment, but after a 12-h fasting and a light, low-fat meal the night. Hemolyzed samples were excluded from the experimental workflow. The freshly whole-blood samples were centrifuged within an hour from collection (3000*g* × 15 min, 4 ℃) to separate plasma. The upper plasma was transferred to a new Eppendorf tube, and then centrifuged (2000*g* × 20 min, 4 ℃) to remove additional cellular fragments. The cleared supernatant was cautiously transferred to another new Eppendorf tube and stored at − 80 ℃. (2) Isolation exosomes from plasma: Exosomes from the supernatant were isolated with exoEasy Maxi kit (Qiagen, Dusseldorf, Germany) according to the manufacturer’s protocol with modifications described in Stranska et al. [6]. The eluates were collected to low protein binding tubes and stored at − 80 ℃. (3) Extraction RNA from exosomes: Exo-miRs were extracted by HiPure Liquid miRNA Kit/HiPure Serum/Plasma miRNA Kit (Megan, China) according to the manufacturer's instructions. The quantity and integrity of exo-miRs yield was assessed by using the Qubit^® ^2.0 (Life Technologies, Carlsbad, USA) and Agilent 2200 TapeStation (Agilent Technologies, Carlsbad, USA) separately. (4) Exo-miRs sequencing: exo-miRs sequencing was performed using Illumina platforms (Illumina, Carlsbad, USA) at Ribobio Co. (Guangzhou, China). Briefly, RNAs were successively ligated with 3′- and 5′-RNA adapter. The adapter-ligated RNAs were then submitted to reverse transcription reaction and amplified with a low-cycle. The PCR products were PAGE-size-selected according to manufacturer's protocol of NEBNext^®^ Multiplex Small RNA Library Prep Set for Illumina (New England BioLabs Inc., Massachusetts, USA). The purified exo-miRs library products were assessed using the Agilent 2200 TapeStation, and then sequenced using an Illumina HiSeq2500 with single-end 50 bp. (5) Sequencing data analysis: The clean reads were acquired after quality control and preprocessing of FASTQ. The miRDeep2 was performed to determine known mature exo-miRs based on miRBase21 (https://www.miRBase.org) and predict novel exo-miRs. The expression of exo-miRs was calculated by reads per million (RPM) values. The differential expression of exo-miRs in subjects with different genotypes of rs1799858 was calculated by edgeR algorithm according to the criteria of |log2 (Fold Change)|≥ 1 and *P* value < 0.05. The online softwares (miRDB, miRTarBase, miRWalk and TargetScan) were performed to predict exo-miRs related targets gene. KOBAS 2.0 software was used to further analysis of GO and KEGG pathway. (6) Validation of top 10 DE-exo-miRs: qRT-PCR array was performed for top 10 DE-exo-miRs in a new verification cohort.

### Statistical analysis

All analysis for baseline characteristics was performed with SPSS version 24 (SPSS, Chicago, USA). Categorical variables were presented as frequencies. Continuous variables were presented as mean ± SD. The differences on continuous variables between the two genotypes (CC vs. TT + CT) of rs1799858 in subjects with increased LDL-C serum concentration (≥ 1.8 mmol/L) were assessed by independent-sample t-test while categorical variables by Chi-square test. The DE-exo-miRs between the two different genotypes was also analyzed with edgeR software. Both GO category and KEGG pathway analyses were evaluated by Chi-square test or Fisher’s exact test. The false discovery rate (FDR) was calculated to adjust the *P* values. If an adjusted *P* value is less than 0.05, the result is considered as significant.

## Results

### Clinical baseline characteristics of study subjects

The clinical features among subjects with different genotypes of *KATP* rs1799858 in this study are shown in Table 1.

**Table 1:** Clinic baseline characteristics of study subjects

	Genotypes of *KATP* rs1799858		*P* value
	*CC*	*TT* + *TC*	
N	7	7	–
Male:female	4:3	4:3	1.000
Age (Y)	47.1 ± 9.0	45.9 ± 8.1	0.671
SBP (mmHg)	112.0 ± 12.7	116.6 ± 9.5	0.226
DBP (mmHg)	72.2 ± 10.2	74.8 ± 7.9	0.401
BMI (kg/m^2^)	24.5 ± 3.8	24.8 ± 2.6	0.793
TRIG (mmol/L)	1.13 ± 0.54	1.08 ± 0.55	0.812
TC (mmol/L)	3.89 ± 0.58	4.21 ± 0.96	0.237
LDL-C (mmol/L)	2.64 ± 0.52	2.86 ± 0.67	0.272
HDL-C(mmol/L)	1.40 ± 0.31	1.27 ± 0.21	0.178
Apo B (mg/dL)	55.1 ± 14.3	54.7 ± 14.8	0.935
Apo A1 (mg/dL)	143.7 ± 15.6	144.6 ± 23.0	0.890
WBC (× 10^9^/L)	9.06 ± 3.84	8.36 ± 3.03	0.550
HGB (g/L)	136.0 ± 19.2	133.5 ± 13.6	0.655
PLT (× 10^9^/L)	220.8 ± 52.6	241.8 ± 52.9	0.243
FBG (mmol/L)	5.27 ± 0.62	5.11 ± 0.60	0.456
P2hBS (mmol/L)	6.07 ± 2.82	6.50 ± 2.53	0.634
HbA1C (%)	5.1 ± 0.9	5.4 ± 0.8	0.357
Cr (μmol/L)	61.3 ± 14.8	64.1 ± 20.6	0.640
BUN (mmol/L)	5.02 ± 2.80	4.78 ± 1.92	0.765
UA (μmol/L)	364.5 ± 82.9	377.1 ± 97.2	0.676
ALT (U/L)	21.7 ± 18.4	25.4 ± 27.8	0.641
AST (U/L)	21.8 ± 9.7	23.7 ± 12.0	0.587
Alb (g/L)	37.6 ± 2.3	38.0 ± 4.7	0.730
Na^+^ (mmol/L)	139.9 ± 3.9	141.8 ± 2.7	0.099
K^+^ (mmol/L)	3.93 ± 0.37	3.88 ± 0.44	0.688
HsCRP (mg/L)	12.2 ± 13.2	13.8 ± 10.8	0.687
MAU (ACR^a^, mg/g)	306.9 ± 89.0	408.8 ± 361.4	0.242
HCY (μmol/L)	14.6 ± 6.9	14.1 ± 3.9	0.782
ACE (U/L)	31.2 ± 17.5	38.2 ± 20.9	0.278
Renin (pg/mL)	26.7 ± 32.4	26.9 ± 23.1	0.981
Ang I (ng/L)	2.82 ± 1.82	1.94 ± 1.29	0.105
Ang II (ng/L)	51.2 ± 37.0	62.4 ± 53.7	0.468
ALD (ng/L)	191.4 ± 101.6	170.0 ± 94.5	0.517

^a^ACR: urinary albumin-to-creatinine ratio

### DE-exo-miRs between different genotypes of *KATP* rs1799858 in subjects with elevated LDL-C (≥ 1.8 mmol/L) serum level

**Fig. 1:**
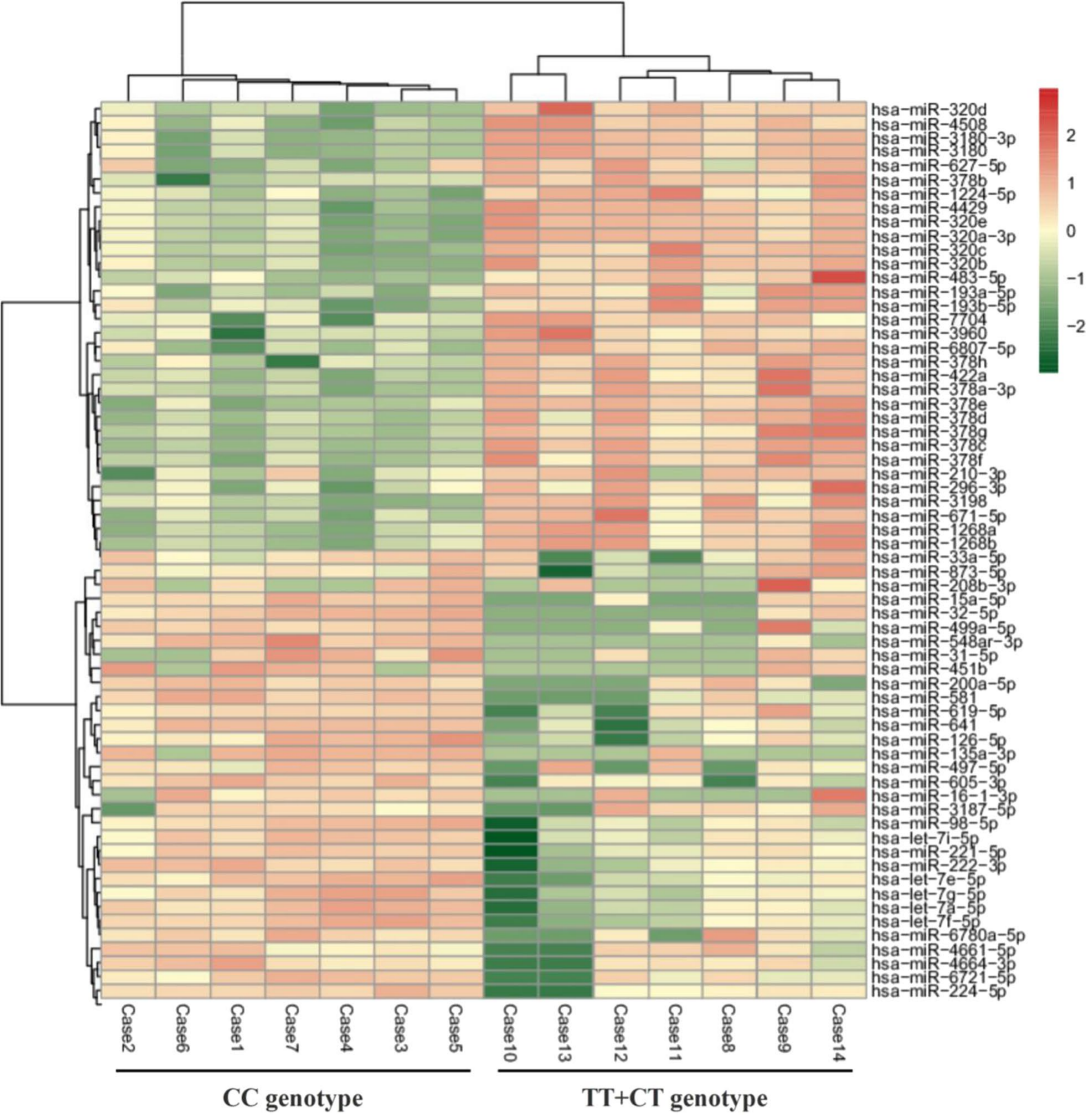
Heatmap of DE-exo-miRs between different genotypes of KATP rs1799858 in subjects with elevated LDL-C (≥ 1.8 mmol/L) serum level

The exo-miRs were analyzed with strict data quality control, and a total of 646 exo-miRs were found. In this study a total of 64 exo-miRs were significantly DE between the two genotypes of rs1799858 with filtering out low-expressing exo-miRs (RPM values < 10), as shown in Additional file 1: Figure S1 and Fig. 1. Among the DE-exo-miRs, 44 exo-miRs were up-regulated and 20 exo-miRs were down-regulated in subjects carrying T-allele (TT + CT) of rs1799858 compared to those with CC genotype. The top 20 up-regulated exo-miRs were miR-483-5p, miR-22-3p, miR-490-3p, miR-378g, miR-320e, miR-6515-5p, miR-31-5p, miR-320b, miR-210-3p, miR-17-3p, miR-320d, miR-6807-5p, miR-378b, miR-378a-3p, miR-497-5p, miR-499a-5p, miR-208b-3p, miR-33a-5p, miR-3611 and hsa-miR-126-5p. The top 20 down-regulated exo-miRs were miR-6780a-5p, miR-619-5p, let-7e-5p, let-7i-5p, let-7g-5p, let-7a-5p, let-7f-5p, miR-641, miR-200a-5p, miR-581, miR-222-3p, miR-605-3p, miR-548ar-3p, miR-221-5p, miR-135a-3p, miR-451b, miR-6721-5p, miR-98-5p, miR-4664-3p and miR-224-5p.

### GO analysis of Enriched biological processes, cellular component and molecular functions regulated by CTGs of top 10 DE-exo-miRs.

**Fig. 2:**
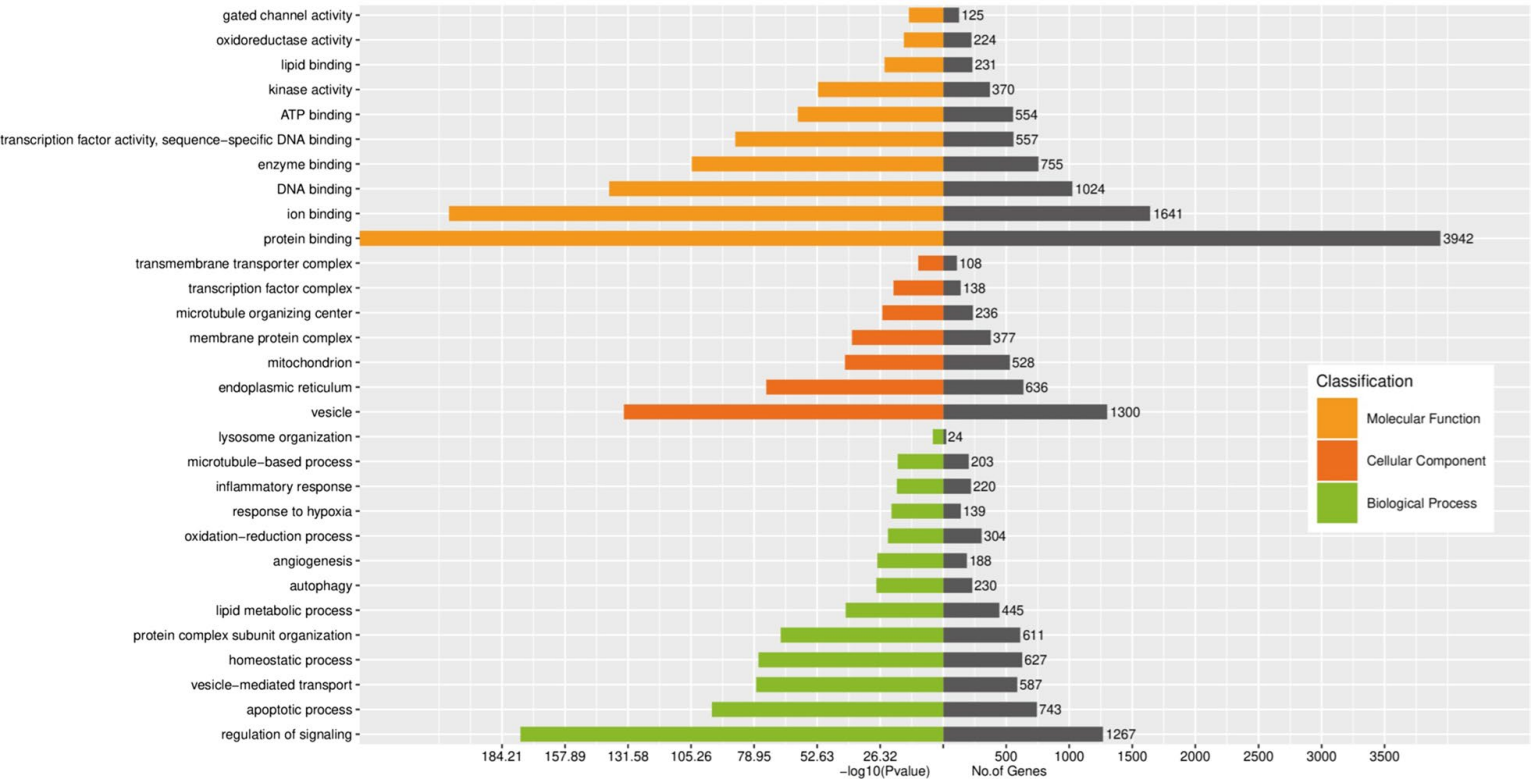
GO analysis of enriched biological processes, cellular component and molecular functions regulated by CTGs of top 10 DE-exo-miRs

GO analysis was used to identify the biological processes, cellular component and molecular functions for top 10 DE-exo-miRs related CTGs. As shown in Fig. 2, the top 10 DE-exo-miRs related CTGs in subjects carrying T-allele (TT + CT) of rs1799858 were obviously linked to regulation of signaling, apoptotic process, vesicle-mediated transport, homeostatic process, protein complex subunit organization, lipid metabolic process, autophagy, angiogenesis, oxidation–reduction process, response to hypoxia, inflammatory response, and microtubule-based process, relating to cellular components such as vesicle, membrane-bounded organelle (e.g., endoplasmic reticulum, mitochondrion, lysosome), membrane protein complex, microtubule organizing center, transcription factor complex, and transmembrane transporter complex. The molecular functions of the top 10 DE-exo-miRs related target genes were correlated with protein binding, ion binding, DNA binding, enzyme binding, sequence-specific DNA binding, ATP binding, transcription factor activity, kinase activity, lipid binding, oxidoreductase activity and gated channel activity.

### KEGG analysis of enrichment pathway regulated by CTGs of top 10 DE-exo-miRs

**Fig. 3:**
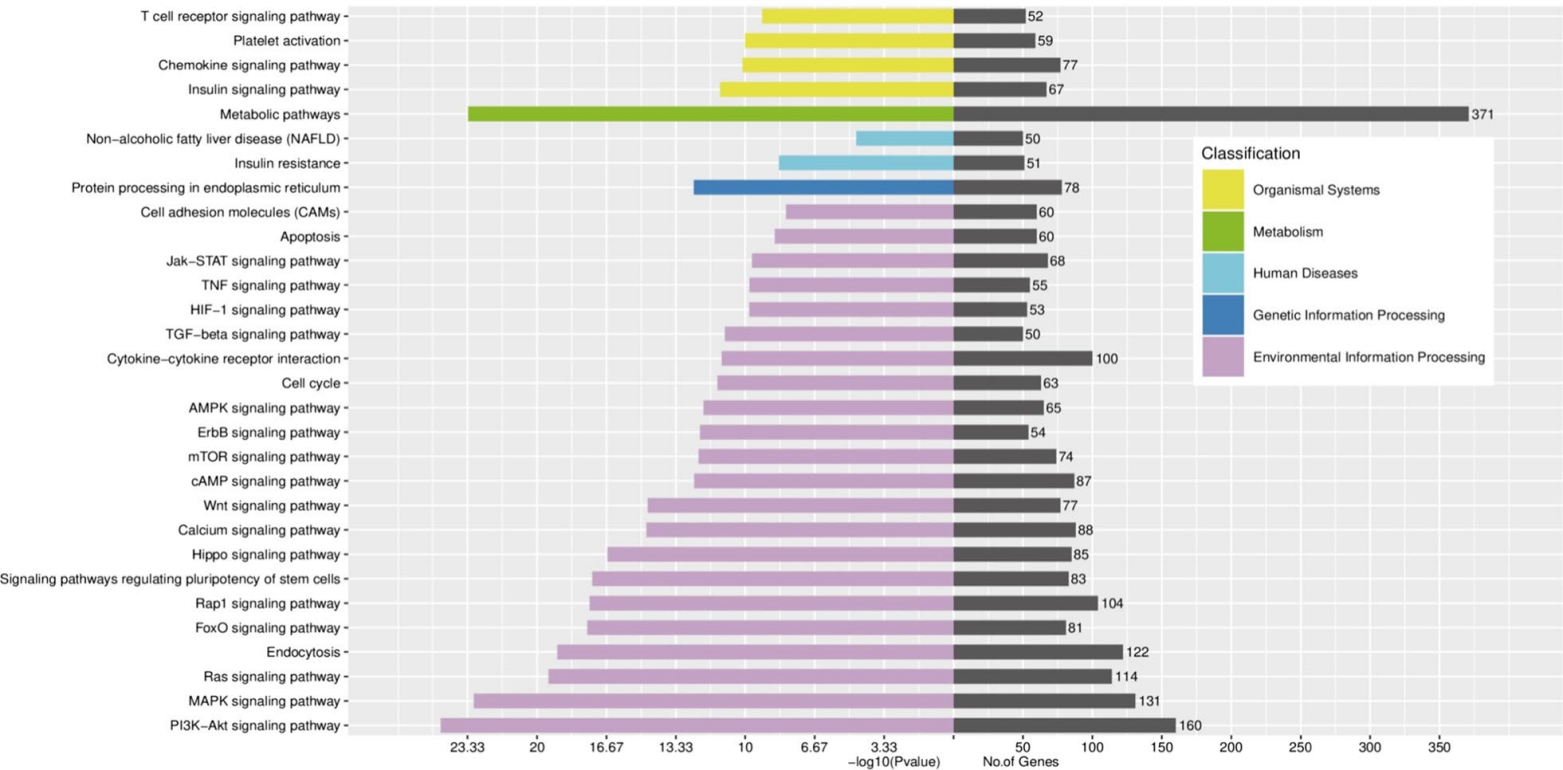
KEGG analysis of enrichment pathway regulated by CTGs of top 10 DE-exo-miRs*. *The top signaling pathways were related to metabolism-related diseases or inflammation including those related to human diseases such as insulin resistance, non-alcoholic fatty liver disease, metabolic/insulin/chemokine/T cell receptor signaling pathways and platelet activation; those related to environmental information processing functions including PI3K-Akt/MAPK/Ras/FoxO/Rap1/Hippo/Wnt signaling pathways; and those related to genetic information/cellular processes such as endocytosis and protein processing in endoplasmic reticulum. Our analysis indicated that these pathways may be involved in the molecular processes from genotype (*KATP* rs1799858) to phenotype (higher LDL-C serum level)

KEGG analysis was also used to identify the for top 10 DE-exo-miRs related CTGs were evidently enriched in 75 pathways. As shown in Fig. 3 and Additional file 1: Figure S2, the top 30 pathways were involved in environmental information processing (e.g., signaling pathways of PI3K-Akt, MAPK and Ras, etc.), genetic information processing (e.g., protein processing in endoplasmic reticulum), human diseases (e.g., insulin resistance and non-alcoholic fatty liver disease), metabolism (e.g., metabolic pathways), and organismal systems (e.g., signaling pathways of insulin, chemokine, platelet activation and T cell receptor).

### Target interactome of top 10 DE-exo-miRs

**Fig. 4:**
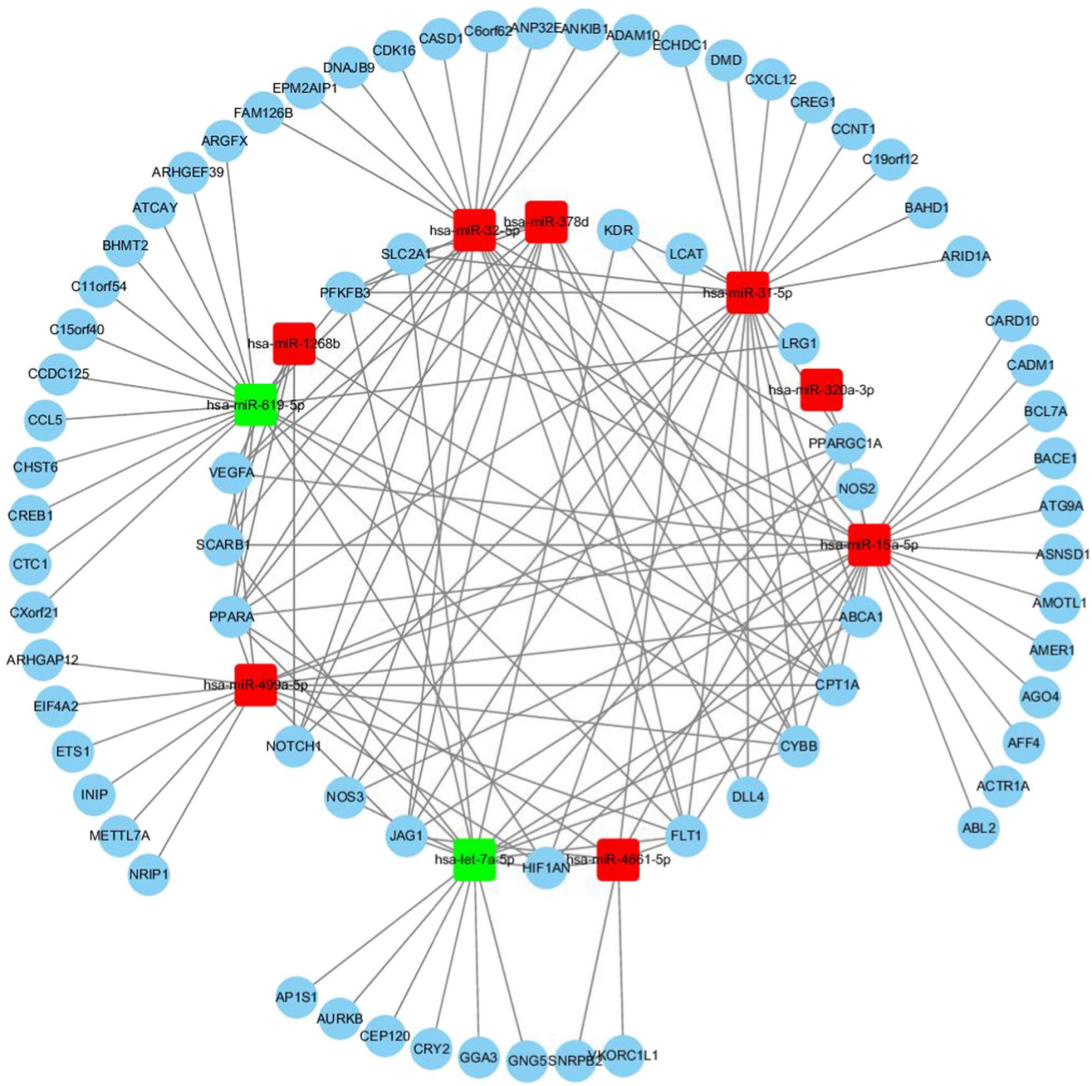
The cross-talk diagram on miRs-gene and gene–gene from top 10 DE-exo-miRs*. *Using combined score > 0.9 as threshold cutoff, 10 exo-miRs and 73 CTGs were included in the internet. Red color represents the up-regulated exo-miRs; Green color represents the down-regulated exo-miRs

There were 1045 CTGs of top 10 DE exo-miRs, and the interactome of these CTGs was determined via STRING online database. As shown in Fig. 4, there were the 10 exo-miRs and 74 CTGs on interaction of exo-miRs/gene and gene/gene by using combined score greater than 0.9 as threshold cutoff. The 10 DE-exo-miRs interacted with target genes, including hypoxia-inducible factor-1α (HIF-1α), nitric oxide synthase (NOS), peroxisome proliferator-activated receptor-α (PPARα), scavenger receptor class B type I (SR-BI), 6-phosphofructo-2-kinase/fructose-2,6-bisphosphatase 2 (PFKFB2), lecithin cholesterol acyltransferase (LCAT), peroxisome proliferator-activated receptor-gamma coactivator-1α (PGC-1α), and ATP binding cassette subfamily A member 1 (ABCA1) so on, which resulted in a complex regulatory network affected by obviously and differently regulated exo-miRs in higher serum LDL-C level (≥ 1.8 mmol/L) subjects with T-allele (TT + CT) of rs1799858.

### qRT-PCR analysis of top 10 DE-exo-miRs

**Fig. 5:**
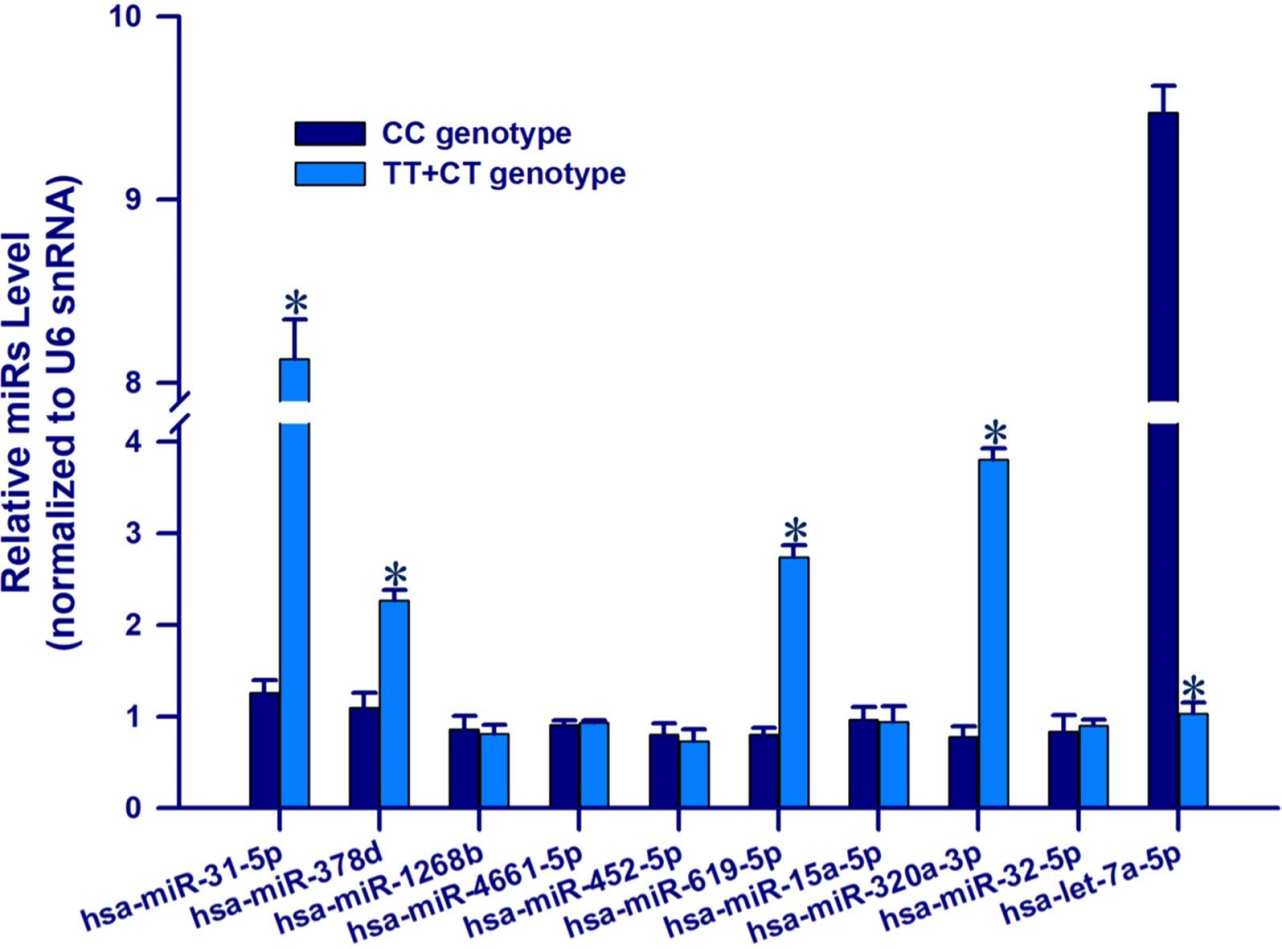
qRT-PCR analysis of top 10 DE-exo-miRs in the validation cohort*. *vs. CC genotype, *P* < 0.05

A total of 50 increased LDL-C levels (≥ 1.8 mmol/L) subjects with T-allele of rs1799858 and 50 same subjects with CC genotype were enrolled to validate the expression of top 10 DE-exo-miRs. Only 5 DE-exo-miRs were successfully validated by qRT-PCR as follows: miR-31-5p (*P* < 0.001), miR-378d (*P* = 0.003), miR-619-5p (*P* = 0.008), miR-320a-3p (*P* < 0.001) and Let-7a-5p (*P* < 0.001), as shown in Fig. 5.

## Discussion

It's well known that genetic or germline variants (e.g., genotype) have a huge impact on the phenotypic landscape of a population. Complex disease (e.g., cardiovascular disease, cancer, etc.) is defined as a phenotype that is caused by many individual gene events, with a significant contribution from environmental factors. Germline variants influence clinic outcomes of complex disease. Recent studies found that functionally mutations of natural killer cells were positively associated with cancer risk [7, 8]. Similarly, in our previous study found that the KATP SNP rs1799858 was associated with increased risk of elevated LDL-C serum concentration (≥ 1.8 mmol/L) and its related macro-/micro-vascular arteriosclerotic events. However, the underlying mechanism of genetic effects on phenotype mediated by genotype-environment interactions remains elusive. exo-miRs, as the leading factor in inducing genetic susceptibility changes, are messengers in the cross-talk between environmental factors and genetic factors. The exo-miRs expression profile and its role of genetic variants on cellular signaling pathways will facilitate our understanding of the relationships between genotype (e.g., *KATP* SNP rs1799858) and phenotype (e.g., LDL-C serum concentration ≥ 1.8 mmol/L and its related ASCVD). This is the only study to reveal a distinct exo-miRs expression in subjects with LDL-C serum concentration ≥ 1.8 mmol/L under specific genetic background of *KATP* polymorphism (rs1799858). (1) We identified the DE-exo-miRs, and respectively screened up-/down-regulating of the top 20 DE-exo-miRs, whose CTGs were identified. (2) GO and KEGG pathway analyses were implemented on those exo-miRs related CTGs. (3) Target interactome network from up-/down-regulating of top 10 DE-exo-miRs was drawn.

Exosomes are secreted by the nucleated cells in response to surrounding environment changes (e.g., increased LDL-C level). Recent research indicated that subjects carrying T allele (TT + CT) of rs1799858 were associated with elevated risk of higher LDL-C (≥ 1.8 mmol/L) level. It was hypothesized that the exo-miRs expression profile varies by genotypes (CC vs. TT + CT) of rs1799858. By next-generation sequencing, there were 64 significantly DE-exo-miRs in increased LDL-C level subjects carrying T allele (TT + CT) of 1,799,858 compared to those with CC genotype. Among 40 DE-exo-miRs (top 20 up-/down-regulated exo-miRs, respectively; Table 2), mi-31-5p was the highest up-regulated exo-miR (approximately 4.5-fold changes), while miR-6780a-5p was the highest down-regulated exo-miR (approximately 2.1-fold changes). There were also 5 exo-miRs families, including miR-378 (e.g., 378a-3p/b/g), miR-320 (e.g., 320b/d/e), miR-208 (e.g., 208b-3p and 499a-5p), Let-7 (e.g., 7a-5p, 7e-5p, 7f-5p, 7g-5p, 7i-5p and miR-98-5p), and miR221/222 (e.g., miR-221-3p and miR-222-5p), which had highly homologous sequence. miR-378 family, originate from the first intron of the PPARγ coactivator 1 beta gene encoding PGC-1β, is a new emerging miR in oxidation/lipid metabolism [9]. The miR-320 family has intrinsic and conserved function for modulation of glucose metabolism under different pathological conditions [10]. As a member of miR-320 family, miR-320b showed the highest expression level among those exo-miRs. The miR-208 family is almost specifically expressed in heart chamber [11] and closely associated with the development of cardiac diseases (e.g., cardiac fibrosis, myocardial hypertrophy, myocardial infarction, and heart failure, etc.). The expression level of miR-208 family increased approximately by threefold. Different from the three up-regulated exo-miRs families, there were also the other two down-regulated exo-miRs families. The Let-7 family, as one of the first-described miR families involving in endothelial cells (ECs) dysfunction and vascular smooth muscle cells (VSMCs) proliferation in the pathogenesis of atherosclerosis, was the one of two down-regulated Exo-miRs family in this study. In contrast, increasing expression level of let-7 family was a protective effect in regulating inflammation in diabetes-related atherosclerosis [12]. miR-221/222 family also involved in the regulation of atherosclerosis [13], because the increased LDL-C level (especially ox-LDL-C) remarkably inhibited miR-221-3p expression in a concentration-dependent and time-dependent manner [14]. Besides the LDL-C, the miR-221/222 family was also associated with low HDL-C phenotype [15]. Similar to the association of miR-221/222 family with dyslipidemia (LDL-C/HDL-C), this phenomenon was also seen on miR-126-5p [16, 17]. In addition, miR-490-3p [18], miR-320b [19], miR-210-3p [20], miR-17-3p [21] and miR-33a-5p [22] were linked to LDL-C metabolism while miR-22-3p [23], miR-31-5p [24], miR-378b [25], and miR-135a-3p [26] were linked to HDL-C metabolism. These findings suggested that the all the 40 DE-exo-miRs may be association with lipid metabolism, especially on LDL-C.

**Table 2:** The top 20 up-/down-regulated DE-exo-miRs between different genotypes of *KATP* rs1799858 in subjects with elevated LDL-C (≥ 1.8 mmol/L) serum level

	miR ID	Genotypes		Fold	*P* value	Up/down
		CC	TT + CT			
1	hsa-miR-483-5p	138.64	1028.68	2.89	1.78E−06	Up
2	hsa-miR-22-3p	9312.58	23,298.12	1.32	4.70E−06	Up
3	hsa-miR-490-3p	3.77	16.62	2.14	6.70E−06	Up
4	hsa-miR-378 g	17.15	52.60	1.62	8.25E−06	Up
5	hsa-miR-320e	170.32	439.14	1.37	1.98E−05	Up
6	hsa-miR-6515-5p	77.16	281.87	1.87	3.10E−05	Up
7	hsa-miR-31-5p	0.61	13.52	4.46	5.05E−05	Up
8	hsa-miR-320b	12,738.79	29,415.44	1.21	0.000119	Up
9	hsa-miR-210-3p	56.64	116.91	1.05	0.000129	Up
10	hsa-miR-17-3p	4.39	15.38	1.81	0.000223	Up
11	hsa-miR-320d	1301.14	3686.95	1.50	0.000526	Up
12	hsa-miR-6807-5p	3.46	7.45	1.11	0.000662	Up
13	hsa-miR-378b	2.62	7.60	1.54	0.000900	Up
14	hsa-miR-378a-3p	6006.05	12,280.17	1.03	0.001102	Up
15	hsa-miR-497-5p	0.59	2.72	2.22	0.001576	Up
16	hsa-miR-499a-5p	26.22	179.18	2.77	0.000640	Up
17	hsa-miR-208b-3p	2.28	20.72	3.18	0.002234	Up
18	hsa-miR-33a-5p	1.44	3.95	1.46	0.006131	Up
19	hsa-miR-3611	0.63	2.01	1.68	0.009428	Up
20	hsa-miR-126-5p	147.59	328.41	1.15	0.009467	Up
21	hsa-miR-6780a-5p	3.57	0.83	− 2.11	0.001143	Down
22	hsa-miR-619-5p	10.44	3.59	− 1.54	0.002449	Down
23	hsa-let-7e-5p	273.17	100.47	− 1.44	0.003298	Down
24	hsa-let-7i-5p	38,517.70	18,945.74	− 1.02	0.014038	Down
25	hsa-let-7g-5p	8191.48	3359.58	− 1.29	0.028204	Down
26	hsa-let-7a-5p	21,006.44	10,351.20	− 1.02	0.030679	Down
27	hsa-let-7f-5p	15,772.96	7041.42	− 1.16	0.041781	Down
28	hsa-miR-641	45.00	16.81	− 1.42	0.008062	Down
29	hsa-miR-200a-5p	3.47	1.54	− 1.17	0.013147	Down
30	hsa-miR-581	6.52	2.88	− 1.18	0.017211	Down
31	hsa-miR-222-3p	2679.15	1329.02	− 1.01	0.022200	Down
32	hsa-miR-605-3p	6.00	2.44	− 1.30	0.023085	Down
33	hsa-miR-548ar-3p	4.47	1.36	− 1.72	0.023611	Down
34	hsa-miR-221-5p	55.87	26.49	− 1.08	0.024990	Down
35	hsa-miR-135a-3p	1.34	0.38	− 1.80	0.026614	Down
36	hsa-miR-451b	3.15	1.08	− 1.54	0.027450	Down
37	hsa-miR-6721-5p	4.49	1.92	− 1.23	0.014197	Down
38	hsa-miR-98-5p	291.28	123.52	− 1.24	0.029034	Down
39	hsa-miR-4664-3p	19.91	9.87	− 1.01	0.038673	Down
40	hsa-miR-224-5p	145.46	61.90	− 1.23	0.042962	Down

Participants with T-allele (TT + CT) of rs1799858 were not only associated with increased risk of higher LDL-C level but also with increased risk of atherosclerosis events, including carotid artery stenosis (CAS) ≥ 50% and new-onset/recurrent acute myocardial infarction (AMI). Synchronously, the data reported in this study indicated that those DE-exo-miRs between the two genotypes (CC vs. TT + CT) of rs1799858 were not only involved in lipid metabolism/dyslipidemia as mentioned above but also played a pivotal role in the occurrence and progression of arteriosclerosis [27, 28], such as miR-22-3p, miR-490-3p, miR-210-3p, miR-497-5p, miR-33a-5p, miR-126-5p, miR-451b, miR-320 family (e.g., miR-320b), miR-208 family (e.g., miR-208b-3p), let-7 family (e.g., let-7i-5p, let-7g-5p, let-7a-5p and let-7f-5p) and miR-221/222 family (e.g., miR-222-3p and miR-221-5p), involving in proliferation and migration of VSMCs (e.g., miR-22-3p [29] and miR-490-3p [18]), ECs dysfunction (e.g., miR-22-3p [30], miR-126 [31], miR-221/222 family [13]), plaque angiogenesis (e.g., miR-126 [32]), apoptosis (e.g., miR-210-3p [33], miR-320d [34]) and macrophage lipid deposition (e.g., miR-210-3p [20]). In particular, miR-483-5p [35], miR-31-5p [36], miR-320b [19] and miR-126 [37] were also closely related to the stenosis degree and unstable phenotype of atherosclerotic plaques, suggesting those exo-miRs may be related to the potential risk of acute vascular events. Indeed, in a four-year prospective study on screening potentially important diagnostic and prognostic biomarkers in acute coronary syndrome resulting from CAS, Gacon et al. [38] found that increased miR-208b-3p level were independently associated with AMI risk, which consistent with the HUNT study by Bye et al. [39] who found that let-7g-5p was associated with fatal future AMI in healthy individuals. The miR-483-5p may be linked to in the early phases of AMI [40]. Under specific genetic background of *KATP* SNP rs1799858, since the accumulation of cardiovascular risk factors (e.g., aging [30], smoking [41],unhealthy diet [26], physical inactivity [42], obesity [43], PM_2.5_ [44], etc.) to the occurrence of ASCVD events and even death [45], these exo-miRs run through the whole cardiovascular event chain, especially such as miR-483-5p, miR-22-3p, miR-31-5p, miR-126, miR-378 family, miR-320 family, let-7 family and miR-221/222 family. However, it is worth mentioning that there were 5 novel exo-miRs (e.g., miR-6515-5p, miR-6807-5p, miR-3611, miR-641 and miR-605-3p), which has no known association with cardiovascular disease, warrant further investigation.

To further investigate the function of exo-miRs under cross-talk status between higher LDL-C level and different genotypes of rs1799858, GO and KEGG analyses were performed for the 1045 CTGs of top 10 DE-exo-miRs in increased LDL-C ≥ 1.8 mmol/L subjects with T-allele (TT + CT) of rs1799858. GO analyses suggested that enrichment of CTGs played crucial roles in biological processes, cellular component and molecular functions (Fig. 2), consistent with a regulatory role on dyslipidemia and related ASCVD [46] for these exo-miRs in the transcription/translation processes [47]. Many differentially regulated KEGG pathways were identified. The results showed that PI3K-Akt signaling pathway, metabolic pathways, and MAPK signaling pathway were the top 3 differentially regulated pathways (Fig. 3). Importantly, PI3K-Akt pathway, which plays an essential role in cellular physiology by regulating growth factor signals during organismal growth and critical cellular processes (e.g., lipid metabolism, glucose homeostasis, protein synthesis, cell proliferation and survival) in normal physiology and morbid conditions (e.g., obesity and T2D) [48]. MAPK pathway, which is known as an important signal transmitter that transmit signals from receptors on the surface to DNA in the nucleus of the cell, is essential in regulating of lipid homeostasis as well as many other cellular processes (e.g., inflammation, cell differentiation, cell division, cell proliferation, motility, apoptosis and stress response) [49]. Both signaling pathways are not only involved in the regulation of lipid homeostasis but also related to atherosclerosis and ASCVD, manifesting the characteristics of synchronous activation [50]. These top 10 exo-miRs were interacted with target genes, forming a network that was influenced by significantly differently regulated exo-miRs in higher LDL-C (≥ 1.8 mmol/L)level patients with T-allele (TT + CT) of rs1799858 (Fig. 4), but only 5 exo-miRs were further comfirmed based on a new verification cohort (Fig. 5). These findings indicated that these DE-exo-miRs could play an important role in increased LDL-C plasma concentration (≥ 1.8 mmol/L) and its related ASCVD by regulating these two pathways.

### Study strengths and limitations

The strength of the study was that this is the first time to characterize the circulating exo-miRs expression profile in biological processes from genotype (*KATP* variant rs1799858) to phenotype (increased LDL-C serum levels), indicating that the potential role of exo-miRs related epigenetic modification on co-evolution of the genetic and environment factors leads to the development of higher LDL-C serum levels and its related ASCVD. There are some limitations in this study. Firstly, the sample size is small so that large-scale, prospective population-based cohort studies will be conducted to confirm the reported results, such as the relationships between these DE-exo-miRs plasma levels and LDL-C exception level related ASCVD events under specific genetic background of KATP variant rs1799858. Secondly, this was only a preliminary bioinformatics analysis so that the possible miss-distance effect and non-specific effect could not be excluded due to lack of validation at the cellular and molecular levels. Finally, due to the validation of DE-exo-miRs by qRT-PCR at the clinical level is incomplete, it is necessary to carry out further functional verification on those 5 validated DE-exo-miRs and its related pathways at the cellular and molecular levels as follows: to study the function of these exo-miRs at the translational or transcriptional levels based on luciferase system or fluorescent microscopy, to identify the specific nucleotide sequences and exo-miRs binding, and to observe its impact on overall functions of signal pathways after mutation. Therefore, results must be interpreted carefully.

## Conclusion

This study firstly indicated that the plasma exo-miRs expression profile bridging the link from genotype (KATP rs1799858) to phenotype (higher LDL-C serum level), and these DE-exo-miRs (especially top 10 DE-exo-miRs) may be potential target intermediates for development of novel diagnosis, prevention, and treatment of LDL-C exception level and its related atherosclerotic vascular disease, warrant further research.

## Supplementary Information

**Additional file 1: Figure S1.** Volcano map of DE-exo-miRs between different genotypes of KATP rs1799858 in subjects with elevated LDL-C (≥1.8 mmol/L) serum level. **Figure S2.** Bubble map for KEGG analysis of enrichment pathway regulated by CTGs of top 10 DE-exo-miRs.

## Data Availability

The datasets used and/or analysed during the current study are available from the corresponding author on reasonable request.

## Abbreviations

ABCA1: ATP binding cassette subfamily A member 1
ABL2: ABL proto-oncogene 2, non-receptor tyrosine kinase
ACE: Angiotensin converting enzyme
ACR: Urinary albumin-to-creatinine ratio
ACTR1A: Actin related protein 1A
ADAM10: ADAM metallopeptidase domain 10
AFF4: AF4/FMR2 family member 4
AGO4: Argonaute RISC component 4
Alb: Albumin
ALD: Aldosterone
ALT: Alanine aminotransferase
AMER1: APC membrane recruitment protein 1
AMI: Acute myocardial infarction
AMER1: APC membrane recruitment protein 1
Ang I/ II: Angiotensin I/II
ANKIB1: Ankyrin repeat and IBR domain containing 1
ANP32E: Acidic nuclear phosphoprotein 32 family member E
AP1S1: Adaptor related protein complex 1 subunit sigma 1
Apo AI: Apolipoprotein AI
Apo B: Apolipoprotein B
ARGFX: Arginine-fifty homeobox
ARHGAP12: Rho GTPase activating protein 12
ARHGEF39: Rho guanine nucleotide exchange factor 39
ARID1A: AT-rich interaction domain 1A
ASCVD: Arteriosclerosis cardiovascular disease
ASNSD1: Asparagine synthetase domain containing 1
AST: Aspartate aminotransferase
ATCAY: ATCAY kinesin light chain interacting caytaxin
ATG9A: Autophagy related 9A
AURKB: Aurora kinase B
BACE1: Beta-secretase 1
BAHD1: Bromo adjacent homology domain containing 1
BCL7A: BAF chromatin remodeling complex subunit 7A
BHMT2: Betaine-homocysteine S-methyltransferase 2
BMI: Body mass index
BUN: Blood urea nitrogen
C6orf62: Chromosome 6 open reading frame 62
C11orf54: Chromosome 11 open reading frame 54
C15orf40: Chromosome 15 open reading frame 40
C19orf12: Chromosome 19 open reading frame 12
CADM1: Cell adhesion molecule 1
CARD10: Caspase recruitment domain family member 10
CAS: Carotid artery stenosis
CASD1: CAS1 domain containing 1
CAD: Coronary atherosclerotic heart disease
CCDC125: Coiled-coil domain containing 125
CCL5: C–C motif chemokine ligand 5
CCNT1: Cyclin T1
CDK16: Cyclin dependent kinase 16
CEP120: Centrosomal protein 120
CHST6: Carbohydrate sulfotransferase 6
CPT-1(CPT1A): Carnitine palmitoyltransferase 1
Cr: Creatinine
CREB1: CAMP responsive element binding protein 1
CREG1: Cellular repressor of E1A stimulated genes 1
CRY2: Cryptochrome circadian regulator 2
CTC1: CST telomere replication complex component 1
CTGs: Candidate target genes
CXCL12: C-X-C motif chemokine ligand 12
CXorf21: Chromosome X open reading frame 21
DBP: Diastolic blood pressure
DE: Differentially expressed
DLL: Delta-like proteins
DMD: Dystrophin
DNAJB9: DnaJ heat shock protein family (Hsp40) member B9
ECHDC1: Ethylmalonyl-CoA decarboxylase 1
ECs: Endothelial cells
eGFR: Estimated glomerular filtration rate
EIF4A2: Eukaryotic translation initiation factor 4A2
EPM2AIP1: EPM2A interacting protein 1
ETS1: ETS proto-oncogene 1, transcription factor
exo-miRs: Exosome-derived microRNAs
FAM126B: Family with sequence similarity 126 member B
FBG: Fasting blood glucose
FDR: False discovery rate
FLT-1 (VEGFR1): Fms related receptor tyrosine kinase 1(vascular endothelial growth factor receptor-1)
GGA3: Golgi associated, gamma adaptin ear containing, ARF binding protein 3
GLUT(SLC2A1): Glucose transporter 1(solute carrier family 2 member 1)
GNG5: G protein subunit gamma 5
GO: Gene Ontology
HbA1C: Glycosylated hemoglobin
HDL-C: High-density lipoprotein cholesterol
HIF-1α: Hypoxia-inducible factor-1α
HGB: Hemoglobin concentration
HsCRP: High-sensitivity C-reactive protein
HTN: Hypertension
INIP: INTS3 and NABP interacting protein
K^+^: Serum potassium
KATP: ATP-sensitive potassium channels
KDR (VEGFR2): Kinase insert domain receptor (vascular endothelial growth factor receptor-2)
KEGG: Kyoto Encyclopedia of Genes and Genomes
LCAT: Lecithin cholesterol acyltransferase
LDL-C: Low-density lipoprotein cholesterol
LRG-1: Leucine rich alpha-2-glycoprotein 1
MAU: Microalbumin in urine
METTL7A: Methyltransferase like 7A
miRs: MicroRNAs
Na^+^: Serum sodium
NOS: Nitric oxide synthase
Notch: Neurogenic locus Notch protein
NOX: NADPH oxidase
NRIP1: Nuclear receptor interacting protein 1
P2hBS: Postprandial blood glucose two hours
PFKFB2(PFK2): 6-Phosphofructo-2-kinase/fructose-2,6-bisphosphatase 2
PGC-1α: Peroxisome proliferator-activated receptor-gamma coactivator-1α
PLT: Platelet count
PPARα: Peroxisome proliferator-activated receptor-α
qRT-PCR: Quantitative real-time polymerase chain reaction
RPM: Reads per million
SBP: Systolic blood pressure
SNP: Single nucleotide polymorphism
SNRPB2: Small nuclear ribonucleoprotein polypeptide B2
SR-BI: Scavenger receptor class B type I
TC: Total cholesterol
T2D: Type 2 diabetes mellitus
TRIG: Triglyceride
UA: Serum uric acid
VEGF: Vascular endothelial growth factor
VKORC1L1: Vitamin K epoxide reductase complex subunit 1 like 1
VSMCs: Vascular smooth muscle cells
WBC: White blood cell count
