# Supplementary material for: Plasma exosome-derived microRNAs expression profiling and bioinformatics analysis under cross-talk between increased low-density lipoprotein cholesterol level and ATP-sensitive potassium channels variant rs1799858
Source: J Transl Med. 2020 Dec 3;18:459. doi: 10.1186/s12967-020-02639-8 (PMC7713329; doi:10.1186/s12967-020-02639-8)
Supplement: Supplementary file 1 — Additional file 1: Figure S1. Volcano map of DE-exo-miRs between different genotypes of KATP rs1799858 in subjects with elevated LDL-C (≥1.8 mmol/L) serum level. Figure S2. Bubble map for KEGG analysis of enrichment pathway regulated by CTGs of top 10 DE-exo-miRs. [file 12967_2020_2639_MOESM1_ESM.docx]

**Additional file**

**Method**

**Sample collection**

Peripheral venous whole-blood samples were collected into anticoagulation tube with EDTA (3 mg/mL) on enrollment, but after a 12-hours fasting and a light, low-fat meal the night. All tubes were centrifuged within an hour from collection at 3000g (Eppendorf 5810R centrifuge, Germany) for 5 minutes at 4˚C to separate plasma and cellular components. Hemolysis was assessed according to the previously reported method, and hemolyzed samples were excluded from the experimental workflow.

**Isolation of exosomes from plasma**

The upper plasma phase was carefully transferred to a new tube with conical bottom without disturbing the intermediate buffy coat layer and centrifuged for 15 min at 3000×g for 10 min at 4 °C to remove additional cellular fragments and debris. The cleared supernatant was carefully transferred to a new tube without disturbing the pellet, which forms a smear along the bottom of the centrifugation tube. Then, the plasma was passed through a 0.22-μm filter to remove larger extracellular vesicles, aliquoted, and stored at -80 °C. Exosomes from prefiltered plasma were isolated with exoEasy Maxi kit (Qiagen, Dusseldorf, Germany; Catalog No. 76064) according to the manufacturer’s protocol with modifications described in Stranska *et al*. [1]. Briefly, 2 ml buffer XBP was added to 2 ml plasma and mixed well by gently inverting the tube five times. Then, the sample/XBP mix was added onto the exoEasy spin column and centrifuged at 500×g for 1 min. The flow-through was discarded and the column was placed back into the same collection tube. After that, 10 ml buffer XWP was added to the column and centrifuged at 3000×g for 5 min to remove any residual buffer from the column. The flow-through along with the collection tube was discarded and the spin column was transferred to a new collection tube. Next, 400 μl buffer XE was added to the membrane and incubated for 1 min, followed by centrifuging at 500×g for 5 min to collect the eluate. Finally, the eluate was re-applied to the exoEasy spin column membrane and incubated for 1 min and then centrifuged at 5000×g for 5 min to collect the eluate.

**Extraction RNA from exosomes**

Exosomal RNA was extracted by HiPure Liquid miRNA Kit/HiPure Serum/Plasma miRNA Kit (Megan, China) according to the manufacturer's instructions. RNA purity was assessed using NanoDrop-1000 (ThermoFisher, California, USA). Each RNA sample had an A260:A280 ratio above 1.8 and A260:A230 ratio above 2.0. The quantity and integrity of Exosomal RNA yield was assessed by using the Qubit®2.0 (Life Technologies, Carlsbad, USA) and Agilent 2200 TapeStation (Agilent Technologies, California, USA) separately.

**Exo-miRs sequencing**

Exo-miRs sequencing was performed using Illumina platforms (Illumina, Carlsbad, USA) at Ribobio Co. (Guangzhou, China). Briefly, RNAs (50ng Exosomal RNA of each sample) were ligated with 3’RNA adapter, and followed by 5’adapter ligation. Subsequently, the adapter-ligated RNAs were subjected to RT-PCR and amplified with a low-cycle. Then the PCR products were size selected by PAGE gel according to instructions of NEBNext Multiplex Small RNA Library Prep Set for Illumina (New England Biolabs, Massachusetts, USA). The purified Exo-miRs library products were evaluated using the Agilent 2200 TapeStation and diluted to 10 pmol/L for cluster generation in situ on the HiSeq2500 single-end flow cell followed by sequencing (1×50 bp) on an Illumina Hiseq 2500 platform. Raw data (raw reads) in fastq format were preprocessed to trim 3’ and 5’adapters, and then, the low-quality reads were filtered out to obtain clean reads. By classifying the clean reads, the components and expression information of all types of small RNAs, including miRs, in the sample can be obtained. miRs expression levels were estimated by the number of reads per million (RPM) using the following formula: RPM=(number of reads mapping to microRNA/number of reads in clean data) ×10^6^.

**Sequencing data analysis**

The raw reads were processed by filtering out containing adapter, poly ’ N’, low quality, smaller than 17nt reads by FASTQC to get clean reads. Mapping reads were obtained by mapping clean reads to reference genome of by BWA. The miRDeep2 was used to identify known mature Exo-miRs based on miRBase21 (www.miRBase.org) and predict novel Exo-miRs. Databases of Rfam12.1 (www.rfam.xfam.org) and pirnabank (www.pirnabank.ibab.ac.in) were used to identify rRNA, tRNA, snRNA, snoRNA and piRNA by BLAST. The Exo-miRs expression were calculated by RPM (reads per million) values [PRM = (number of reads mapping to miRNA/ number of reads in Clean data) ×10^6^]. The expression levels were normalized by RPM, RPM is equal to (number of reads mapping to miRs/number of reads in clean data) ×10^6^. Exo-miRs differential expression in subjects with between two genotypes of rs1799858 was calculated by edgeR algorithm according to the criteria of | log2 (Fold Change) | ≥1 and *P* value < 0.05. miRDB, miRTarBase, miRWalk and TargetScan were used to predict targets gene of selected Exo-miRs. KOBAS was used to further Gene Ontology (GO) and KEGG (Kyoto Encyclopedia of Genes and Genomes) pathway analysis.

**Validation of top 10 DE Exo-miRs**

Total RNA was extracted according to the methods mentioned above. cDNA was synthesized from 1.0 μg of total RNA using using the TaqMan microRNA Reverse Transcription Kit (Thermo Fisher Scientific) and individual TaqMan miRNA assays (Thermo Fisher Scientific) for tissue and plasma samples. Quantitative real-time PCR was performed by the TaqMan Fast Universal PCR Master Mix (2x) (Thermo Fisher Scientific) on a Quantstudio 7 Flex Real-Time PCR System (Thermo Fisher Scientific) according to the manufacturer’s protocol for TaqMan miRNA assays with minor modifications. Negative control reactions contained no cDNA templates. For quantitative results, expression of miRNA was expressed as fold change using the 2^−ΔΔCt^ method. Differences in expression of top 10 Exo-miRs between the two genotypes of *KATP* rs1799858 were analyzed by independent-sample t-test.

**Results**

**Clinic baseline characteristics of study subjects**

As shown in Table 1, there were no significant differences on gender, age, Blood pressure levels (SBP and DBP), blood lipid levels (TRIG, TC, LDL-C, HDL-C, Apo B and Apo AI), WBC, HGB, PLT, blood glucose (FBG, P2hBS, HbA1C), renal function (Cr, BUN, UA), liver function (ALT, AST, Alb), blood electrolytes (serum sodium and potassium) and the activation of RAAS (ACE, renin, Ang I/II and ALD) (all P>0.05).

**DE-exo-miRs between different genotypes of *KATP* rs1799858 in subjects with elevated LDL-C (≥1.8 mmol/L) serum level**

As shown in Additional file 1: Figure S1 and Figure 1, Exo-miRs were analyzed with strict data quality control, and a total of 646 Exo-miRs were found. After filtering out low-expressing Exo-miRs (RPM values < 10 in both groups) by using *P* < 0.05 as threshold cutoff, a total of 64 Exo-miRs were significantly DE between the two genotypes of rs1799858.

**Figure legends**

Figure S1. Volcano map of DE-exo-miRs between different genotypes of KATP rs1799858 in subjects with elevated LDL-C (≥1.8 mmol/L) serum level.

Figure S2. Bubble map for KEGG analysis of enrichment pathway regulated by CTGs of top 10 DE-exo-miRs.

**
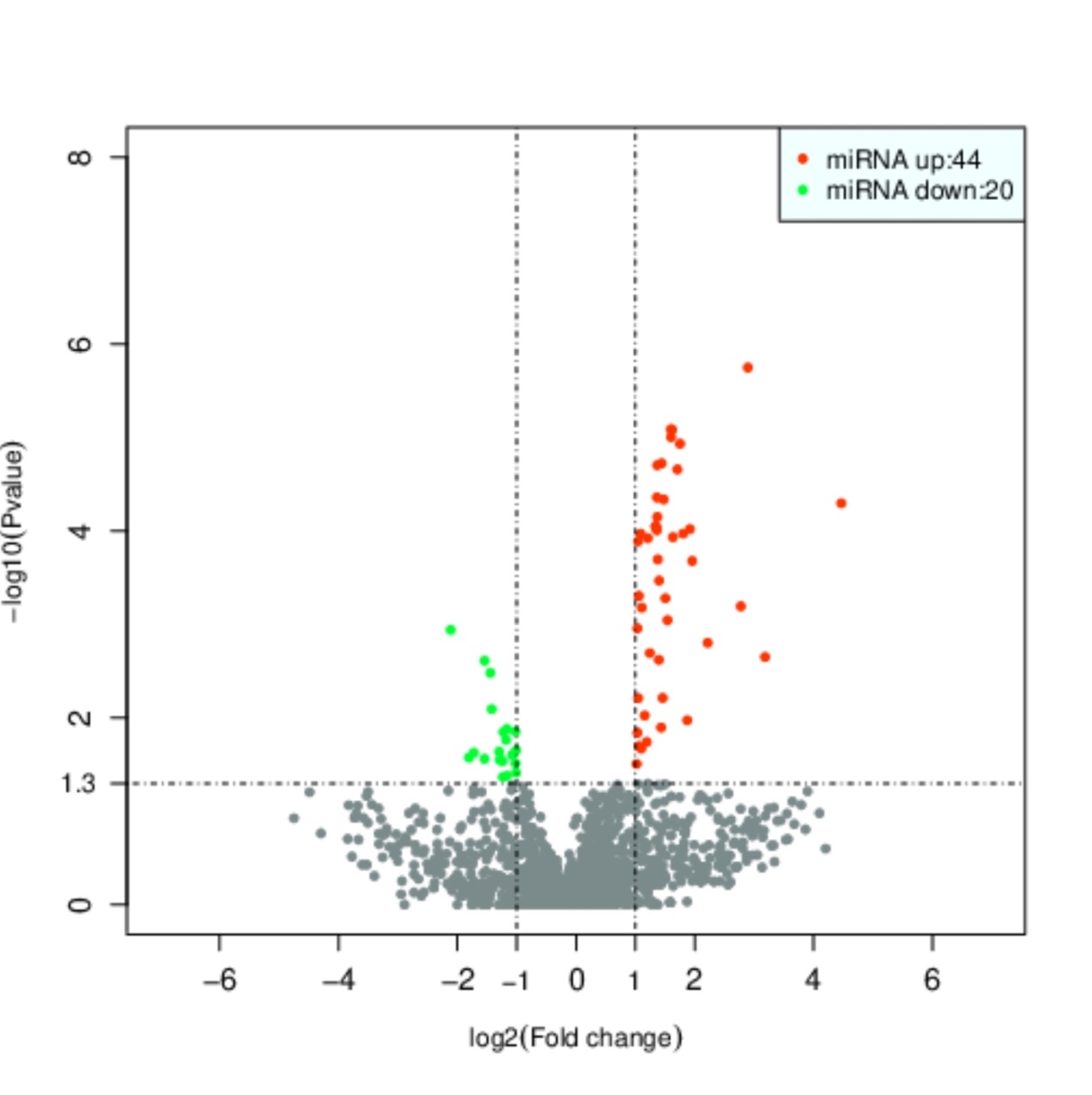
**

**Figure S1. Volcano map of DE-exo-miRs between different genotypes of KATP rs1799858 in subjects with elevated LDL-C (≥1.8 mmol/L) serum level.**

**
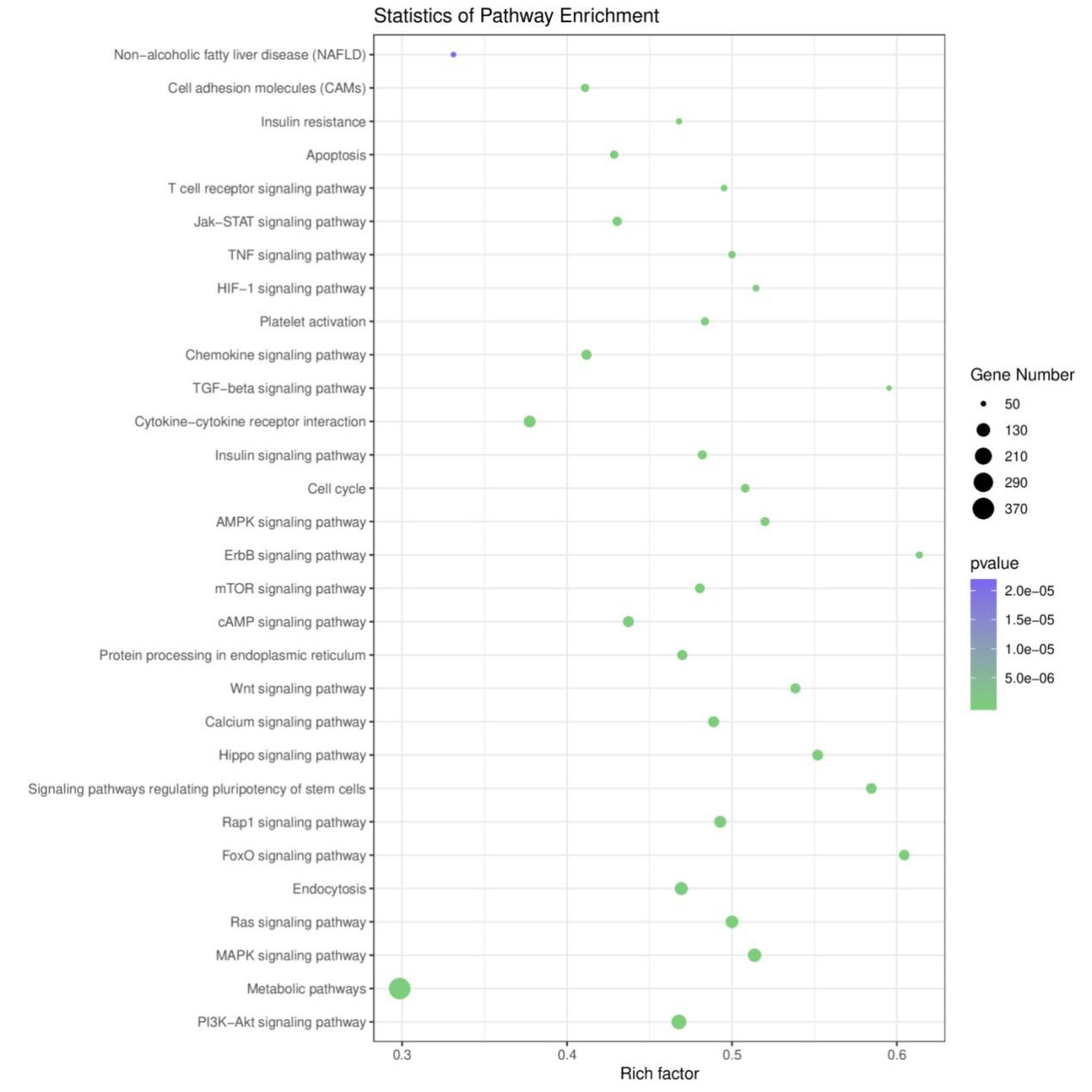
**

**Figure S2. Bubble map for KEGG analysis of enrichment pathway regulated by CTGs of top 10 DE-exo-miRs.**

**References**

1. Stranska R, Gysbrechts L, Wouters J, Vermeersch P, Bloch K, Dierickx D, Andrei G, Snoeck R: **Comparison of membrane affinity-based method with size-exclusion chromatography for isolation of exosome-like vesicles from human plasma.** *J Transl Med* 2018, **16:**1.
